# No association between low-dose aspirin use and breast cancer outcomes overall: a Swedish population-based study

**DOI:** 10.1186/s13058-018-1065-0

**Published:** 2018-11-20

**Authors:** Gabriella Frisk, Sara Ekberg, Elisabet Lidbrink, Sandra Eloranta, Malin Sund, Irma Fredriksson, Mats Lambe, Karin E. Smedby

**Affiliations:** 1 Department of Medicine Solna, Division of Clinical Epidemiology, Karolinska Institutet and Karolinska University Hospital, SE-171 76 Stockholm, Sweden; 2 Department of Oncology–Pathology, Karolinska Institutet, Stockholm, Sweden; 3 Department of Surgical and Perioperative Sciences, Umeå University, Umeå, Sweden; 4 Department of Molecular Medicine and Surgery, Karolinska Institutet, Stockholm, Sweden; 5 Department of Breast and Endocrine Surgery, Karolinska University Hospital, Stockholm, Sweden; 6 Department of Medical Epidemiology and Biostatistics, Karolinska Institutet, Stockholm, Sweden

**Keywords:** Aspirin, Breast cancer, Sweden, Registers

## Abstract

**Background:**

Results from previous studies indicate that use of low-dose aspirin may improve breast cancer prognosis. We evaluated aspirin use and breast cancer outcomes in relation to clinical characteristics as well as dose and duration of aspirin use.

**Methods:**

We used information from the Regional Breast Cancer Quality-of-Care Registries in three Swedish regions to identify 21,414 women diagnosed with a first stage I–III breast cancer between 1 April 2006 and 31 December 2012. The cohort was further linked to nationwide registers to retrieve information about dispensing low-dose aspirin before and after breast cancer diagnosis, comorbidity and causes of death. In a separate analysis, we investigated time to breast cancer death among 621 women with stage IV disease at diagnosis. Associations were evaluated using a multivariable Cox proportional hazards model.

**Results:**

Among women with stage I–III breast cancer, 2660 (12.4%) used low-dose aspirin shortly before breast cancer diagnosis and 4091 (19.1%) were users during follow-up. Women were followed for a median of 3.8 years after diagnosis. There was no association between aspirin use and breast cancer-specific death in multivariable analyses (use before diagnosis: hazard ratio (HR) 0.93, 95% confidence interval (CI) 0.77–1.12; use after diagnosis: HR 1.00, 95% CI 0.74–1.37). Similarly, aspirin use was not associated with risk of first recurrence/metastases in a subgroup of stage I–III breast cancer patients (HR 0.97, 95% CI 0.86–1.10). However, in analyses stratified by stage, an inverse association between low-dose aspirin use after diagnosis and breast cancer death was found for women with stage I tumors (HR 0.53, 95% CI 0.29–0.96). Among women with stage IV disease at diagnosis, aspirin use was not associated with time to breast cancer death (HR 0.91, 95% CI 0.67–1.23).

**Conclusion:**

In this large population-based cohort study there was no evidence that low-dose aspirin use before or after breast cancer diagnosis is associated with a reduced risk of adverse outcomes overall in breast cancer. However, a potential benefit was noted among women with stage I tumors, warranting further investigation.

**Electronic supplementary material:**

The online version of this article (10.1186/s13058-018-1065-0) contains supplementary material, which is available to authorized users.

## Background

Breast cancer is the most common malignancy among women in high-income countries. About 8000 new cases are diagnosed each year in Sweden [1], 255,000 cases in the USA [2] and 55,000 cases in the UK [3]. Early detection of breast cancer with mammographic screening and more effective adjuvant treatment has gradually improved the prognosis in breast cancer [4, 5]. However, a considerable number of women die from the disease [3]. Hence, additional cost-effective therapies are still needed. Several studies have indicated that low-dose aspirin use around the time of a breast cancer diagnosis may reduce the risk of both breast cancer-specific and all-cause mortality [6–9], but results are inconsistent. A few studies have also reported no associations between aspirin use after breast cancer diagnosis and breast cancer deaths [10, 11]. In a recent meta-analysis, pooled results found evidence of a reduction in breast cancer-specific death following aspirin use versus no use: RR of 0.73 (95% CI, 0.54–0.98, *p* = 0.04) [12]. However, there was heterogeneity among the included studies and following exclusion of one deviant study, the significant association between postdiagnostic aspirin use and breast cancer-specific mortality disappeared.

There are several plausible biological mechanisms of action for a potential beneficial effect of aspirin use in breast cancer initiation and progression, involving inflammation, hormonal alterations and platelet inhibition [13, 14]. Aspirin irreversibly inhibits cyclooxygenase (COX)-1 and COX-2 which are crucial for synthesis of prostaglandins involved in cellular migration and proliferation. Prostaglandins are present at elevated levels in breast cancer tissue, where they are believed to also stimulate angiogenesis and inhibit apoptosis, and inhibition of COX-1 and COX-2 has reduced growth of breast cancer cell lines [15]. Prostaglandins further stimulate aromatase activity, which subsequently increase estrogen levels, and lower levels have been reported in postmenopausal aspirin users compared to nonusers [16]. Aspirin could perhaps also inhibit platelet-induced adhesion of circulating tumor cells from initiating metastases [17, 18].

Because of the mixed results from previous studies and a lack of randomized trials, large observational studies remain important to understand whether aspirin has the potential of altering breast cancer prognosis, and, if so, among which subgroups of women. Two randomized trials on aspirin use are ongoing, one in the USA and one in the UK, to evaluate aspirin use and disease-free survival in women with early-stage breast cancer [19], but results will be not be available for many years (preliminarily in 2026). In the present study, we have used detailed clinical information from Swedish population-based breast cancer quality-of-care registers and the national drug prescription register to address the possible associations between low-dose aspirin use and outcomes in subgroups of women with breast cancer. We tested the hypothesis that aspirin use is associated with improved breast cancer-specific outcomes overall or in certain clinical subgroups of breast cancer.

## Methods

### Study population and setting

We used a cohort study design to investigate the association between low-dose aspirin use and risk of breast cancer-specific death as primary outcome. Aspirin use was assessed through records of drug dispensing shortly before and after breast cancer diagnosis as well as during the entire follow-up. The study population was identified through three regional breast cancer quality-of-care registers linked with nationwide health-care registers under the acronym BcBaSe Sweden (the Stockholm-Gotland, Uppsala-Örebro and Northern regions) and included all women diagnosed with a primary invasive breast cancer during the period 1 April 2006–31 December 2012. The starting date was given by the availability of information on drug dispensing of low-dose aspirin from July 2005 (see later) plus a period of 9 months for prediagnostic exposure assessment. Compared to the mandated reporting to the National Swedish Cancer Register [20], the completeness of the breast cancer quality-of-care registries is in excess of 90% [21]. Furthermore, health care in Sweden is tax-funded with specialized care in oncology being accessible to all residents. By means of record linkage to the National Swedish Cancer Register, we excluded women with a record of an earlier breast cancer diagnosis while women with other previous cancer diagnoses were not excluded (Additional file 1: Figure S1). Women with breast cancer stage I–III at diagnosis were included in the main analysis, whereas women with stage IV disease at diagnosis were analyzed separately. Among women with stage I–III breast cancer diagnosed in the Stockholm-Gotland region, we also analyzed time to local recurrence or distant metastases as a secondary outcome. Information available from the regional quality-of-care breast cancer registers included age, region, date and clinical TNM classification of disease at diagnosis, estrogen receptor (ER) status, human epidermal growth factor receptor (HER2) status and neoadjuvant and intended adjuvant treatments (chemotherapy, radiotherapy, endocrine therapy and/or trastuzumab). The concordance between register records of intended adjuvant treatment and actually administered treatment has been shown to be high (90%) [22]. Based on the variables in the regional quality-of-care register, we classified the breast cancers as luminal (ER^+^, HER2^–^/HER2^+^), nonluminal HER2 (ER^–^, HER2^+^) or ER^–^HER2^–^.

### Classification of aspirin use

The Swedish Prescription Register (SPR) has recorded all filled prescriptions at Swedish pharmacies from 1 July 2005 onward [23]. From the SPR, we ascertained any dispensing of low-dose aspirin to assess aspirin use. Our definition of dispensed aspirin was limited to daily doses of 75 or 160 mg (ATC codes B01AC06, B01AC30 and B01AC56). These doses are available only by prescription and represent 90% of all aspirin sold nationally (by prescription or over the counter) [24]. We did not consider exposure during 90 days before or after breast cancer diagnosis since women may have taken more aspirin before diagnosis due to local symptoms or may have been told to avoid aspirin in conjunction with surgery. Aspirin use (yes/no) was assessed during a 6-month period before breast cancer diagnosis (≥ 1 dispensing from 9 to 3 months before breast cancer diagnosis) and during a 6-month period shortly after breast cancer diagnosis (≥ 1 dispensing 3–9 months after breast cancer diagnosis). In addition, to estimate cumulative use, aspirin use was assessed during the entire follow-up post diagnosis (from 3 months after breast cancer diagnosis and onward) as a time-varying exposure. For each dispensing, SPR contains information on the date, number of packages, package size and milligrams (mg) per tablet dispensed. There is also a text variable with the doctor’s prescription (e.g., “1 tablet a day”). We calculated the prescribed daily dose as the number of prescribed tablets per day times the number of mg per tablet. The prescribed daily dose was divided into ≤ 75 mg per day and > 75 mg per day. The cumulative number of days of aspirin use was calculated as the number of tablets dispensed divided by the number of tablets prescribed per day and updated at every dispensing. Women who changed doses during follow-up (*n* = 258, 6.3% of aspirin users) were excluded from the time-varying analyses. The cumulative number of days of use was grouped as follows: none, < 6 months, 6 months–2 years and > 2 years.

### Ascertainment of comorbidity and other covariates

Using the national registration numbers assigned to all Swedish residents [25], the cohort was linked to nationwide registers including the SPR (described earlier), the National Patient Register (NPR) and the longitudinal integration database for health insurance and labor market studies (LISA). In the NPR, the Swedish National Board of Health and Welfare has compiled data on individual hospital discharges. Each record contains medical data including diagnoses at discharge according to the International Classification of Diseases (ICD) and dates of admission and discharge. Since 2001, this register also records visits to nonprimary outpatient care, with an estimated proportion of register dropouts of only 2% [26]. The NPR was used to assess comorbidity at diagnosis as well as locoregional recurrence or distant metastases during follow-up (see later). The LISA database includes information on highest achieved educational level (≤ 9 years, 10–12 years, > 12 years), used as a proxy for socioeconomic status [27]. Comorbidity was assessed based on NPR records during 5 years before diagnosis and was classified into two major groups: diseases for which low-dose aspirin use is recommended (cardiovascular, inflammatory and cerebrovascular disorders); and diseases where aspirin may be counterindicated (peptic ulcer disease, chronic liver failure and asthma) (Additional file 1: Table S1). Since the NPR is confined to records of hospital admissions and/or nonprimary outpatient visits, and not visits to the general practitioner, the assessment of comorbidity is likely to mainly reflect severe disorders requiring specialized care. We also assessed dispensing of other nonsteroidal anti-inflammatory drugs (NSAIDs), statins and metformin (through ATC codes M01A, C10AA and A10BA02) from the SPR as potential confounders covering prescriptions from both primary and specialized care. Use of these drugs were assessed as ever/never use during the same time windows as aspirin (dose and duration were not considered).

### Outcome

Women diagnosed with stage I–III breast cancer were followed from 9 months after breast cancer diagnosis until death or 31 December 2012, whichever occurred first. In the analysis of stage IV patients, and in analyses of risk of first recurrence/metastasis among stage I–III patients in the Stockholm-Gotland region (42% of the cohort), patients were followed from the date of diagnosis. For classification of breast cancer-specific death, the cohort was linked to the Cause of Death register [28]. When breast cancer was recorded as the main underlying cause of death, it was regarded as breast cancer specific. Date of recurrence/metastasis was defined as the date of the first record in the NPR with a diagnosis code of locoregional recurrence or distant metastasis (ICD C77–C79). As breast cancer patients are routinely followed in specialist (nonprimary) care, visits due to recurrence/metastases should be recorded in the NPR. This complementary analysis, however, was restricted to women residing in the Stockholm-Gotland region because the completeness of recurrence and metastasis coding in the NPR has been found to be particularly high in this region.

### Statistical analysis

We analyzed the association between low-dose aspirin use, risk of breast cancer-specific death and time to recurrence/metastasis (Stockholm-Gotland region) using a Cox proportional hazards model yielding hazard ratios (HRs) with 95% confidence intervals (CIs) as measures of association. When we classified aspirin use we used a 180-day lag period in order to disregard changes in prescription patterns during the last 6 months prior to death as these may reflect changes in personal drug administration routines due to end-of-life care [29–31]. The main models were stratified on tumor stage to allow for different baseline hazards and to account for deviations from the proportional hazards assumption. We used a crude model with no adjustments, a second model adjusted for age at diagnosis, stage, year of diagnosis, region, education level and comorbidity before diagnosis, and a third model adjusted for the same variables plus statin, metformin and NSAID use as well as oncological treatment (neoadjuvant/adjuvant therapy in four categories: chemotherapy, radiotherapy, endocrine therapy and/or trastuzumab) to illustrate the effect of stepwise addition of potential confounding factors. We adjusted for confounders on the basis of a priori selection of factors suggested in other studies to be potential confounders in analyses of aspirin use and breast cancer progression/death. In analyses of aspirin use after breast cancer diagnosis, the models were additionally adjusted for low-dose aspirin use before diagnosis. The underlying timescale was time since breast cancer diagnosis. The proportional hazards assumption was formally tested using the Grambsch and Therneau test [32]. Adjusted graphical predictions of breast cancer-specific survival according to aspirin use after diagnosis (3–9 months) were obtained from a flexible parametric survival model [33] with five degrees of freedom to model the baseline hazard function with a restricted cubic spline. For comparison, this adjusted survival function was presented together with survival estimates obtained using the Kaplan–Meier method.

We further analyzed aspirin use before and after diagnosis among subgroups of women by clinical and tumor characteristics and oncological treatment and risk of breast cancer-specific death. Thus, second sets of models were fitted for stage of disease and ER status, HER2 status, subtype and oncological treatment. Interaction tests were performed with likelihood ratio tests.

All analyses were performed with Stata 14 software (StataCorp. 2015. Stata Statistical Software: Release 14; StataCorp LP, College Station, TX, USA).

## Results

### Clinical characteristics

The cohort consisted of 21,414 women with breast cancer diagnosed in stage I–III (Table 1). The median age at breast cancer diagnosis was 63 years (range 19–102) with a median follow-up of 3.8 years (range 0.75–7.75). Overall, 2660 women (12.4%) were treated with low-dose aspirin before breast cancer diagnosis. Aspirin users were older at diagnosis (median age 75 years, range 31–102), and more often diagnosed with stage II–III than stage I tumors compared with nonusers (Additional file 1: Table S2). Considering the entire follow-up after breast cancer diagnosis, 4091 women (19.1%) used low-dose aspirin.

**Table 1:** Characteristics of cohorts, women with breast cancer stage I–III and IV, diagnosed 2006–2012

	Stage I–III	Stage IV
	(*n* = 21,414)	(*n* = 621)
Age at diagnosis		
< 40 years	883 (4.1)	17 (2.8)
40–49 years	3114 (14.5)	63 (10.1)
50–59 years	4597 (21.5)	109 (17.6)
60–69 years	6546 (30.6)	132 (21.3)
70–79 years	3679 (17.2)	154 (24.8)
≥ 90 years	2595 (12.1)	146 (23.6)
Age (years), median (range)	63 (19–102)	68 (29–97)
Region		
Stockholm-Gotland	9068 (42.4)	235 (37.8)
Uppsala-Örebro	8868 (41.4)	263 (42.4)
North	3478 (16.2)	123 (19.8)
Education		
< 10 years	5367 (25.1)	227 (36.6)
10–12 years	8682 (40.5)	230 (37.0)
> 12 years	7119 (33.2)	147 (23.7)
Missing	246 (1.2)	17 (2.7)
Stage		
I	12,546 (58.6)	na
II	7879 (36.8)	na
III	989 (4.6)	na
IV	na	621
HER2 status		
Positive	2407 (11.2)	50 (8.1)
Negative	15,848 (74.0)	147 (23.7)
Missing	3159 (14.8)	424 (68.3)
ER status		
Positive	17,514 (81.8)	166 (26.7)
Negative	2907 (13.6)	60 (9.7)
Missing	993 (4.6)	395 (63.6)
Breast cancer subtype		
Luminal (ER^+^, HER2^–^/HER2^+^)	15,529 (72.5)	141 (22.7)
Nonluminal HER2 (ER^–^, HER2^+^)	857 (4.0)	34 (5.5)
ER^–^HER2^–^	1739 (8.1)	18 (2.9)
Missing	3289 (15.4)	428 (68.9)
Neoadjuvant^a^/adjuvant breast cancer treatment		
Chemotherapy	8401 (39.2)	97 (15.6)
Endocrine therapy	16,160 (75.5)	141 (22.7)
Radiotherapy	15,036 (70.2)	82 (13.2)
Trastuzumab	1858 (8.7)	21 (3.4)
Aspirin		
Before breast cancer diagnosis (from 9 to 3 months)	2660 (12.4)	100 (16.1)
After breast cancer diagnosis (3–9 months)	2813 (13.1)	na
During entire follow up	4091 (19.1)	na
Aspirin treatment, duration (total, at end of follow-up)		
< 6 months	485 (2.3)	na
6 months–2 years	1552 (7.2)	na
> 2 years	2054 (9.6)	na
Dose of aspirin (total, at end of follow-up)		
< 75 mg/day	24 (0.1)	na
75 mg/day	3486 (16.3)	na
> 75 to < 160 mg/day	258 (1.2)	na
160 mg/day	223 (1.0)	na
> 160 mg/day	100 (0.5)	na

Data presented as *n* (%) unless indicated otherwise. *ER* estrogen receptor, *HER2* human epidermal growth factor receptor 2, *na* not applicable

^a^Neoadjuvant radiotherapy, 41 women (0.2%); neoadjuvant chemotherapy, 891 women (4.1%); neoadjuvant endocrine therapy, 691 women (3.2%)

The majority of the women (12,546; 58.6%) were diagnosed with stage I breast cancer, 7879 women (36.8%) had stage II and 989 women (4.6%) had stage III disease. The most common clinical subtype was the luminal subtype (ER^+^, HER2^–^/HER2^+^), recorded in 15,529 women (72.5%), whereas 857 women (4%) had nonluminal HER2 (ER^–^, HER2^+^) and 1739 women (8.1%) had ER^–^HER2^–^ breast cancer.

### Low-dose aspirin use before diagnosis in stage I–III breast cancer and risk of breast cancer-specific death

There were no associations between aspirin use from 9 to 3 months before breast cancer diagnosis and risk of breast cancer death when adjusted for age, stage of primary breast cancer, education, region, year of primary diagnosis and comorbidity before breast cancer diagnosis (HR 0.92, 95% CI 0.77–1.09) (Table 2). Further adjustment for use of other medications (statins, metformin and NSAIDs) before breast cancer diagnosis and neoadjuvant/adjuvant oncological treatments (chemotherapy, endocrine therapy, radiotherapy and/or trastuzumab) did not change the result (HR 0.93, 95% CI 0.77–1.12). The dose of aspirin (≤ 75 or > 75 mg/day) before diagnosis did not modify the null association. In patient subgroups by clinical characteristics, however, reduced risks of breast cancer death were suggested among women with ER^+^ tumors (HR 0.74, 95% CI 0.57–0.97) and among those with intended endocrine treatment (HR 0.75, 95% CI 0.59–0.96) (Additional file 1: Table S3).

**Table 2:**
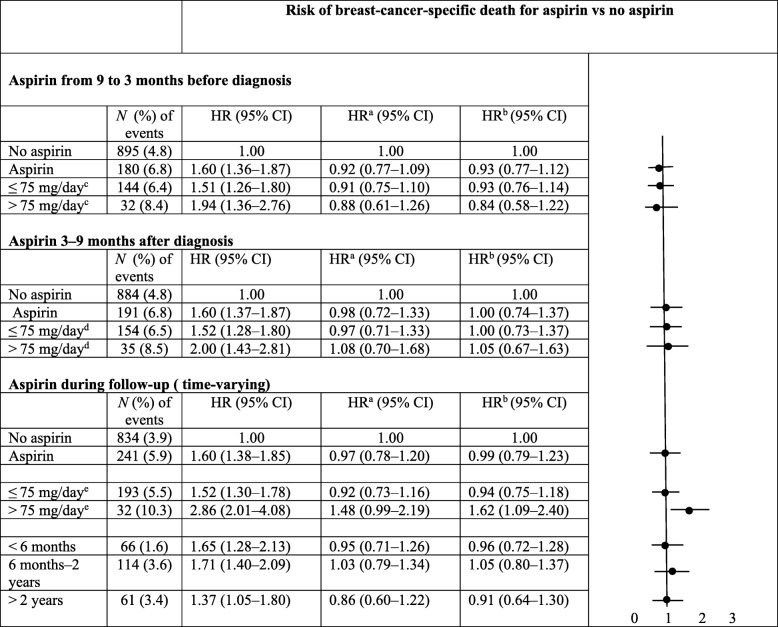
Aspirin use and risk for breast cancer-specific death in women with stage I–III breast cancer

*CI* confidence interval, *HR* hazard ratio

^a^Adjusted for age at diagnosis, stage, year of diagnosis, region, education level and comorbidity (including inflammatory diseases, heart disease, cerebrovascular disease, atherosclerotic disease, thromboembolic venous disease, hyperlipidemia, hypertension, peptic ulcer, liver disease, asthma) before diagnosis

^b^Adjusted for age at diagnosis, stage, year of diagnosis, region, education level, comorbidity before diagnosis, statin use, metformin use and nonsteroidal anti-inflammatory drug use (excluding aspirin) (yes/no) during the same time interval as aspirin (before or after diagnosis or during follow-up) and adjuvant oncological treatment (chemotherapy, endocrine therapy, trastuzumab and radiotherapy). In the analyses of aspirin after diagnosis and during follow-up, we additionally adjusted for prediagnostic aspirin use

^c^20 patients had missing information on dose

^d^16 patients had missing information on dose

^e^ 258 patients (6.3% of aspirin users) changed dose during follow-up (excluded)

### Low-dose aspirin use after diagnosis in stage I–III breast cancer and risk of breast cancer-specific death

**Fig. 1:**
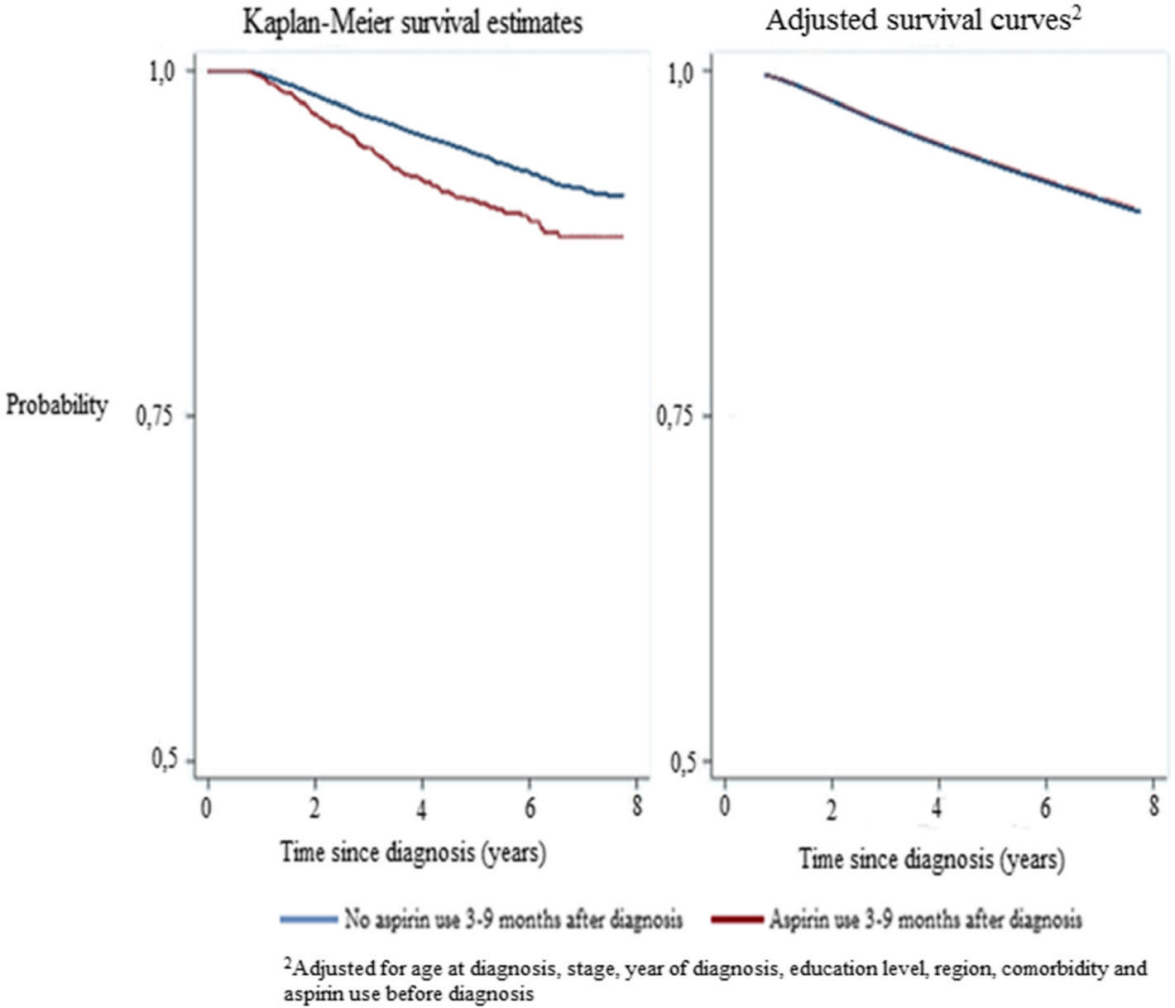
Survival of women with breast cancer stage I–III and medication with aspirin after diagnosis. Blue line, no aspirin use 3–9 months after diagnosis; red line, aspirin use 3–9 months after diagnosis. ^2^Adjusted for age at diagnosis, stage, year of diagnosis, education level, region, comorbidity and aspirin use before diagnosis

Aspirin use during the period 3–9 months after breast cancer diagnosis did not affect the risk of breast cancer-specific death in a full multivariate model also adjusting for prediagnostic aspirin use (HR 1.00, 95% CI 0.74–1.37) (Table 2). This is also shown graphically in an adjusted survival curve and compared with the univariable Kaplan–Meier method (Fig. 1). When aspirin use over the entire follow-up post diagnosis was considered, the result was similar (HR 0.99, 95% CI 0.79–1.23) (Table 2). In general, the dose and duration of aspirin use after diagnosis was not associated with breast cancer-specific death. However, in the subgroup of women treated with aspirin > 75 mg daily during the entire follow-up, an increased risk of breast cancer-specific death was observed (HR 1.62, 95% CI 1.09–2.40). In subgroups of patients defined by clinical and tumor characteristics (stage, ER status, HER2 status, breast cancer subtype and oncological treatment), aspirin use after diagnosis was associated with a reduced risk of breast cancer-specific death among women with stage I tumors (HR 0.53, 95% CI 0.29–0.96) (Table 3). There was also a borderline significantly increased risk among women with stage III tumors.

**Table 3:**
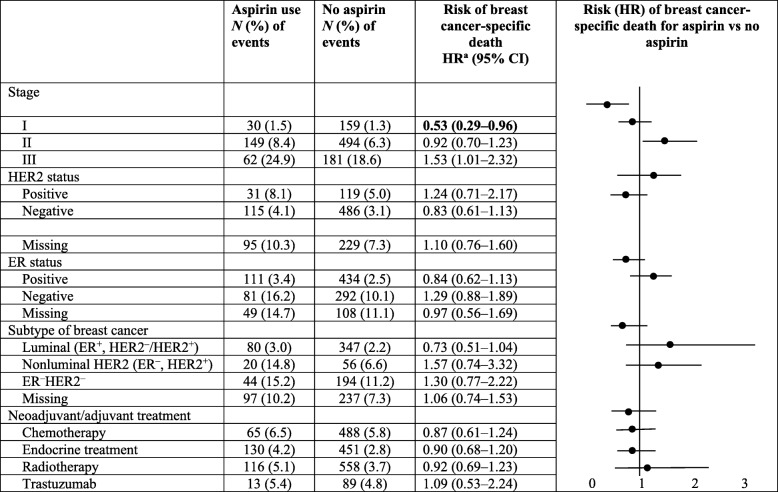
Aspirin use after diagnosis (time-varying) and risk of breast cancer-specific death within clinical subgroups

*CI* confidence interval, *ER* estrogen receptor, *HER2* human epidermal growth factor receptor 2, *HR* hazard ratio

^a^Adjusted for age, stage, education, comorbidity (including inflammatory diseases, heart disease, cerebrovascular disease, atherosclerotic disease, thromboembolic venous disease, hyperlipidemia, hypertension, peptic ulcer, liver disease, asthma), year of diagnosis, region and aspirin use before diagnosis

Bold data represent significant values

### Low-dose aspirin use before diagnosis in breast cancer stage I–III and time to first recurrence/metastasis

In the Stockholm-Gotland regional cohort, there were 9226 women with stage I–III disease, of which 1048 women (11.4%) were aspirin users before breast cancer diagnosis (Additional file 1: Table S4). During follow-up, 2800 women not using aspirin (34.2%) and 347 women treated with aspirin (33.1%) had a record of a first recurrence or distant metastasis. Aspirin use was not associated with risk of recurrence/metastases in univariate analysis (HR 0.98, 95% CI 0.88–1.09) or in a model adjusted for age at diagnosis, stage, year of diagnosis, region, educational level and comorbidity before breast cancer diagnosis (HR 0.97, 95% CI 0.86–1.10).

### Low-dose aspirin use before diagnosis in stage IV patients and time to breast cancer-specific death

In a separate analysis, we investigated aspirin use before breast cancer diagnosis and time to breast cancer death among 621 women with stage IV disease at diagnosis. Time to breast cancer death was not significantly different for aspirin users with stage IV disease (*n* = 61) when compared to nonusers (*n* = 334) (HR 0.91, 95% CI 0.67–1.23) in adjusted analyses.

## Discussion

In this large Swedish population-based cohort study we found no evidence that low-dose aspirin use before or after breast cancer diagnosis reduces the risk of breast cancer-specific death in breast cancer patients overall. There were no indications of dose-response by dose or duration of aspirin use. However, among women with stage I tumors, aspirin use after diagnosis was associated with a reduced risk of breast cancer death. We also found a possible reduced risk in women with ER^+^ tumors who were treated with low-dose aspirin before breast cancer diagnosis. Even though these associations could also have arisen by chance, further subgroup-specific investigations in larger datasets are warranted to confirm or refute these findings. Aspirin did not reduce the risk of metastases among stage I–III breast cancer patients, nor prolong the time to breast cancer-specific death in stage IV disease.

Our results corroborate findings from some, but not all, earlier studies. HRs for aspirin use assessed before diagnosis have shown no association in two studies [11, 34] and an increased risk of breast cancer death in one study [8]. Results for aspirin use assessed after diagnosis (and risk of breast cancer death) range from no association in five studies [10, 11, 34–36] to a protective association in three studies [6–8]. In a meta-analysis of five randomized trials of daily aspirin (≥ 75 mg) versus placebo for prevention of cardiovascular events in the UK, a reanalysis of risk for cancer metastases indicated a lower rate of metastases in breast cancer patients although the association was not statistically significant [37].

From the Iowa Women’s health study, Blair et al. [7] reported that breast cancer patients using aspirin after diagnosis were at a significantly lower risk for breast cancer death compared with nonusers. The aspirin exposure in this study was self-reported through a questionnaire and focused on current use of aspirin, and modifications in risk by clinical factors such as tumor size were found. Similarly, Holmes et al. [6] reported current aspirin use to be associated with a substantially decreased risk of breast cancer death, with an adjusted RR of 0.36 (0.24–0.54) for aspirin users 6–7 days per week when compared with nonusers from the Nurses’ Health Study. However, past aspirin use was not associated. Results did not differ appreciably when stratified by stage, BMI, menopausal status or ER status.

In a Swedish register-based study with a nested case–control design, aspirin use after breast cancer diagnosis was not associated with risk of breast cancer death, except when aspirin use had been terminated close to death/end of follow-up. However, clinical information such as tumor stage at diagnosis was not available. It cannot be excluded that this finding reflects confounding by indication and/or reverse causation, since patients may stop taking aspirin and other drugs due to a worsening general condition in the end-of-life period. Also, patients admitted to hospital or palliative care in Sweden generally do not receive their drugs by prescription and are therefore not recorded in the prescription registry. In our study, aspirin exposure was handled as a time-varying exposure with a 180-day lag to avoid bias due to change in medication close to death [29–31]. This may explain why our results differ from the previous Swedish study and the two American studies.

In a recent cohort study from the Scottish Cancer Register including 15,140 stage I–III breast cancer patients, Mc Menamin et al. [36] reported a HR of 0.92 (95% CI 0.75–1.14) for breast cancer death among women using aspirin after breast cancer diagnosis compared with nonusers. They also examined low-dose aspirin use before breast cancer diagnosis with no association with cancer-specific mortality (adjusted HR 0.95, 95% CI 0.81–1.11). With a median follow-up of 4 years, the Scottish study also lagged medication use after diagnosis by 6 months to avoid bias due to change in medication. They had detailed data on clinical factors such as stage and ER status, but could not find any protective effect on breast cancer-specific mortality in these subgroups. However, the HR point estimate was lower among stage I patients than in other groups (HR 0.74, 95% CI 0.35–1.54).

Other epidemiological studies have also reported no association for aspirin use after diagnosis and risk of breast cancer death. In an Irish cohort study of 4540 women aged 50–80 years with stage I–III breast cancer who were nonaspirin users before diagnosis, aspirin initiation after diagnosis meant no reduction of breast cancer-specific mortality. Aspirin exposure was identified from linked national prescription data, and the analysis was adjusted for clinical characteristics. There was no evidence of effect modification by tumor size, lymph node status or ER status, but subgroup-specific results were not presented [10].

In summary, several recent studies do not provide support for an association between aspirin use and breast cancer progression overall, but they have had limited power to investigate potential differences across patient subgroups. Our study represents one of the largest so far, and therefore the finding of a potential association among stage I patients, and perhaps also in the ER^+^ group, warrants further investigation. Since aspirin inhibits prostaglandins which in turn leads to inhibition of angiogenesis and stimulation of apopotosis, as well as to lower estrogen levels through an impact on aromatase activity, there are several biologically plausible mechanisms that could explain a putative association [13, 14, 17, 18]. It is perhaps not unfeasible that biological effects, if at all present, could be confined to early-stage ER^+^ tumors given putative anti-hormonal effects. Alternatively, early-stage tumors with slow progression may be more susceptible than aggressive ones. In a few patient subgroups (users of high-dose aspirin (> 75 mg) and patients with stage III tumors), moderately increased risks of breast cancer deaths were noted. Although we cannot exclude true subgroup-specific increased risks, we believe that the most likely explanations for these results include chance and/or potential residual confounding by the indications for aspirin use.

Strengths of our study include the use of information from a large population-based unselected cohort of women with breast cancer identified from regional prospective quality-of-care registers. These databases provide information on patient and tumor characteristics as well as treatment intentions allowing for detailed subgroup analyses and adjustments. By use of data on drug dispensing from a national register, we avoided potential bias by self-reported data. Swedish health-care register data are generally of high quality and completeness. Data available from the National Patient Register allowed for adjustments for comorbidity. Several limitations need mentioning. Since aspirin is sold over the counter in Sweden, we cannot exclude that some aspirin users were misclassified as nonusers. However, low-dose aspirin represents 90% of all aspirin sold, and this form is only available by prescription [24]. Another limitation is that the prescription register provides information on dispensed drugs only, without taking into account patient adherence and actual use. Confounding by indication is a challenge in pharmaco-epidemiological studies [38] and might also have affected our study since the prescribed dose of aspirin differs by indication. However, a majority of women (85.2%) were prescribed 75 mg daily dose of aspirin, the standard dose for prevention of heart disease in Sweden. Only a small fraction (6.3%) of women had their dose changed during follow-up. In the analysis of the association between aspirin use before breast cancer diagnosis and breast cancer mortality, there is potential selection bias/collider stratification bias when stratifying by tumor stage, which may change the association between aspirin use before diagnosis and breast cancer mortality in either direction. However, by adjusting for potential confounders between stage and breast cancer mortality this bias should have been reduced. Still, the results for aspirin use before breast cancer diagnosis should be interpreted assuming that there is no residual confounding. We did not have data on potentially important confounders such as BMI, smoking habits or physical activity. However, findings from a few earlier studies have not found evidence of any large confounding of the association between aspirin and breast cancer progression by these factors [6, 7, 39].

## Conclusions

In this large population-based cohort of women with breast cancer, we found no strong evidence of a protective effect of low-dose aspirin use before or after breast cancer diagnosis, either regarding risk of breast cancer death or time to first recurrence/metastasis. However, in subgroups of women with more favorable tumor characteristics, such as stage I disease, aspirin use is potentially associated with a favorable outcome. Further subgroup-specific studies of a potential benefit of aspirin among early-stage breast cancer women are warranted.

## Additional file

Additional file 1: **Table S1.** Number of patients with comorbidity and medications among stage I–III and ICD10 codes of comorbidities. **Table S2** Clinical and breast cancer characteristics of aspirin and nonaspirin users with breast cancer stage I–III. **Table S3** Aspirin use from 9 to 3 months before breast cancer diagnosis and risk of breast cancer-specific death. **Table S4** Clinical characteristics of breast cancer women with stage I–III in Stockholm/Gotland and incidence of recurrence. **Figure S1** Flow diagram of included study participants (DOCX 67 kb)

## Abbreviations

ATC: Anatomic Therapeutic Chemical classification
CI: Confidence interval
ER: Estrogen receptor
HER2: Human epidermal growth factor receptor 2
HR: Hazard ratio
ICD: International Classification of Diseases
LISA: Longitudinal integration database for health insurance and labor market studies
NPR: National Patient Register
NSAID: Nonsteroidal anti-inflammatory drug
SPR: Swedish Prescription Register
