# Supplementary material for: No association between low-dose aspirin use and breast cancer outcomes overall: a Swedish population-based study
Source: Breast Cancer Res. 2018 Nov 20;20:142. doi: 10.1186/s13058-018-1065-0 (PMC6247765; doi:10.1186/s13058-018-1065-0)
Supplement: Supplementary file 1 — Table S1. Number of patients with comorbidity and medications among stage I–III and ICD10 codes of comorbidities. Table S2 Clinical and breast cancer characteristics of aspirin and nonaspirin users with breast cancer stage I–III. Table S3 Aspirin use from 9 to 3 months before breast cancer diagnosis and risk of breast cancer-specific death. Table S4 Clinical characteristics of breast cancer women with stage I–III in Stockholm/Gotland and incidence of recurrence. Figure S1 Flow diagram of included study participants (DOCX 67 kb) [file 13058_2018_1065_MOESM1_ESM.docx]

**Table S1: Number of patients with comorbidity and medications among stage I-III and ICD10 codes of comorbidities**

|  | **Stage I-III** | |  |
| --- | --- | --- | --- |
|  | **Patients with comorbidity before breast cancer diagnosis**  **N (%)** | **Patients with comorbidity after breast cancer diagnosis**  **N (%)** | **ICD 10-code**  **(from 1998)** |
| **Disorders associated with use of aspirin** | 4 547 (21.2) | 5 150 (24.1) |  |
| Inflammatory diseases | 496 (2.3) | 262 (1.2) | M05, M06, M123, M08, M09, M13, M45, L405, M07, M320, M321, M328, M329, M460, M461, M468, M469, M02, M358, M359 |
| Ischemic heart disease | 926 (4.3) | 773 (3.6) | I20, I21, I22, I23, I24, I25 |
| Other cardiovascular disease | 1 372 (6.4) | 1 322 (6.2) | 147-149, 151-152, I70-I79 |
| Cerebrovascular disease | 533 (2.5) | 610 (2.9) | I60-I69 |
| Other atherosclerotic disease | 218 (1.0) | 239 (1.1) | G45, K55.0-K55.1 |
| Congestive heart disease | 540 (2.5) | 877 (4.1) | I11.1, I11.0, I11.3, I42-143, I50 |
| Heart valve disease | 238 (1.1) | 235 (1.1) | I05-I08, I33-I39 |
| Thromboembolic venous disease | 313 (1.5) | 1 209 (5.7) | I26, I80-I82, 187.0 |
| Other (Hyperlipidemia, Hypertonia) | 2 760 (12.9) | 4 079 (19.0) | E78, I10, I15 |
| **Disorders associated with decreased use of Aspirin** | 1 469 (6.9) | 1 561 (7.3) |  |
| Asthma | 499 (2.3) | 656 (3.1) | J45, J46 |
| Liver disease | 96 (0.5) | 132 (0.6) | K70-77 |
| Peptic ulcer | 960 (4.5) | 960 (4.5) | K20-K30 |
| **Other medications** |  |  |  |
| NSAID | 2 424 (11.3) | 8 658 (40.4) |  |
| Paracetamol | 2 889 (13.5) | 10 667 (49.8) |  |
| Statins | 2 841 (13.3) | 4 179 (19.5) |  |
| Metformin | 689 (3.2) | 1 289 (6.0) |  |

Diagnosis: First diagnosis, a patient has their first record *either* before or after breast cancer diagnosis

Medication: Before breast cancer diagnosis (9-3 months) and after breast cancer diagnosis (from 3 months after), a patient can have records from both periods.

**Table S2: Clinical and breast cancer characteristics of aspirin and non-aspirin users with breast cancer stage I-III**

|  | **Aspirin use *before* diagnosis**  **(9-3 months before)**  N (%) | **Aspirin use *after* diagnosis**  **(3-9 months after)**  N (%) | No Aspirin use N (%) |
| --- | --- | --- | --- |
|  | 2 660 | 2 813 | 18 331 |
| **Stage** |  |  |  |
| I | 1 344 (50.5) | 1 415 (50.3) | 10 983 (59.9) |
| II | 1 153 (43.4) | 1 221 (43.4) | 6 552 (35.7) |
| III | 163 (6.1) | 177 (6.3) | 796 (4.3) |
| **HER2 status** |  |  |  |
| Positive | 234 (8.8) | 243 (8.6) | 2 138 (11.7) |
| Negative | 1 792 (67.4) | 1 908 (67.8) | 13 765 (75.1) |
| Missing | 634 (23.8) | 662 (23.5) | 2 428 (13.3) |
| **ER status** |  |  |  |
| Positive | 2 055 (77.3) | 2 187 (77.8) | 15 122 (82.5) |
| Negative | 322 (12.1) | 342 (12.2) | 2 529 (13.8) |
| Missing | 283 (10.6) | 284 (10.1) | 680 (3.7) |
| **Type of cancer** |  |  |  |
| Luminal (ER+, HER2-/HER2+) | 1 728 (65.0) | 1 840 (65.4) | 13 519 (73.8) |
| Non-luminal HER2 (ER-, HER2+) | 84 (3.2) | 93 (3.3) | 757 (4.1) |
| ER-HER2- (ER-, HER2-) | 198 (7.4) | 200 (7.1) | 1 516 (8.3) |
| Missing | 650 (24.4) | 680 (24.2) | 2 539 (13.9) |
| **Neoadjuvant/Adjuvant treatment** |  |  |  |
| Chemotherapy | 499 (18.8) | 524 (18.6) | 7 806 (42.6) |
| Endocrine treatment | 1 975 (74.3) | 2 097 (74.6) | 13 871 (75.7) |
| Radiotherapy | 1 338 (50.3) | 1 443 (51.3) | 13 453 (73.4) |
| Trastuzumab | 125 (4.7) | 134 (4.8) | 1 706 (9.3) |

**Table S3. Aspirin use 9-3 months *before* breast cancer diagnosis and risk of breast cancer-specific death**

|  | **Aspirin use**  **N (%) of events** | **No aspirin N (%) of events** | **Risk of breast cancer-specific death**  **HR^1^ (95% CI)** |
| --- | --- | --- | --- |
| **Stage** |  |  |  |
| I | 26 (1.9) | 163 (1.5) | 0.99 (0.62,1.56) |
| II | 114 (9.9) | 529 (7.9) | 0.98 (0.78,1.23) |
| III | 40 (24.5) | 203 (24.6) | 0.72 [0.50,1.05] |
| **HER 2 status** |  |  |  |
| Positive | 19 (8.1) | 131 (6.0) | 0.77 [0.45,1.32] |
| Negative | 88 (4.9) | 513 (3.7) | 1.01 [0.79,1.30] |
| Missing | 73 (11.5) | 251 (9.9) | 0.80 [0.60,1.07] |
| **ER-status** |  |  |  |
| Positive | 75 (3.7) | 470 (3.0) | 0.74 [0.57,0.97] |
| Negative | 63 (19.6) | 310 (12.0) | 1.21 [0.89,1.65] |
| Missing | 42 (14.8) | 115 (16.2) | 0.83 [0.56,1.22] |
| **Subtype of breast cancer** |  |  |  |
| Luminal (ER+, HER2-/HER2+) | 57 (3.3) | 370 (2.7) | 0.77 [0.57,1.04] |
| Non-luminal HER2 (ER-, HER2+) | 12 (14.3) | 64 (8.3) | 0.94 [0.44,2.02] |
| ER-HER2- (ER-, HER2-) | 37 (18.7) | 201 (13.0) | 1.29 [0.86,1.94] |
| Missing | 74 (11.4) | 260 (9.9) | 0.80 [0.61,1.06] |
| **Neoadjuvant/Adjuvant treatment** |  |  |  |
| Chemotherapy | 41 (8.2) | 512 (6.5) | 0.91 [0.64,1.30] |
| Endocrine treatment | 90 (4.6) | 491 (3.5) | 0.75 [0.59,0.96] |
| Radiotherapy | 78 (5.8) | 596 (4.4) | 0.91 [0.70,1.19] |
| Trastuzumab | 5 (4.0) | 97 (5.6) | 0.38 [0.14,1.04] |

**^1^**Adjusted for age, stage, education, comorbidity (including inflammatory diseases, heart disease, cerebrovascular disease, atherosclerotic disease, thromboembolic venous disease, hyperlipidemia, hypertension, peptic ulcer, liver disease, asthma), year of diagnosis, region and aspirin use before diagnosis

CI: Confidence Interval, HR: Hazard Ratio

**Table S4. Clinical characteristics of breast cancer women with stage I-III in Stockholm/Gotland and incidence of recurrence**

|  | **Stage I-III patients**  **N=9 226** | Recurrence/metastasis | |
| --- | --- | --- | --- |
|  | **N (%)** | N (%) | Rate per 100 person-years  (95% CI) |
| **Age at diagnosis, years** |  |  |  |
| <30 | 58 (0.6) | 27 (0.3) | 18 (12-26) |
| 30-39 | 396 (4.3) | 183 (2.0) | 17 (15-20) |
| 40-49 | 1 469 (15.9) | 580 (6.2) | 14 (13-15) |
| 50-59 | 1 958 (21.2) | 689 (7.5) | 11 (11-12) |
| 60-69 | 2 702 (29.3) | 773 (8.4) | 9 (8-10) |
| 70-79 | 1 511 (16.4) | 521 (5.6) | 13 (12-14) |
| 80-89 | 924 (10.0) | 302 (3.3) | 13 (12-15) |
| ≥90 | 208 (2.3) | 72 (0.8) | 20 (16-25) |
| **Median age (range)** | 61 (21-102) |  |  |
| **Education, years** |  |  |  |
| <10 | 1 848 (20.0) | 618 (6.7) | 12 (11-13) |
| 10-12 | 3 537 (38.3) | 1 239 (13.4) | 12 (11-13) |
| >12 | 3 693 (40.0) | 1 234 (13.4) | 11 (11-12) |
| Missing | 148 (1.6) | 56 (0.6) | 16 (12-21) |
| **Stage** |  |  |  |
| I | 5 229 (56.7) | 1085 (11.7) | 6 (6-6) |
| II | 3 509 (38.0) | 1672 (18.2) | 20 (19-21) |
| III | 488 (5.3) | 390 (4.2) | 76 (69-84) |
| **HER 2 status** |  |  |  |
| Positive | 878 (9.5) | 399 (4.3) | 20 (18-22) |
| Negative | 6 224 (67.5) | 2060 (22.3) | 13 (12-13) |
| Missing | 2 124 (23.0) | 688 (7.5) | 8 (8-9) |
| **ER-status** |  |  |  |
| Positive | 7 503 (81.3) | 2 387 (25.9) | 11 (10-11) |
| Negative | 1 190 (12.9) | 540 (5.9) | 17 (16-18) |
| Missing | 533 (5.8) | 220 (2.4) | 21 (18-24) |
| **Type of breast cancer** |  |  |  |
| Luminal (ER+, HER2-/HER2+) | 6 116 (66.3) | 1 994 (21.6) | 12 (12-13) |
| Non-luminal HER2 (ER-, HER2+) | 316 (3.4) | 177 (1.9) | 30 (26-34) |
| ER-HER2- (ER-, HER2-) | 645 (7.0) | 273 (3.0) | 17 (15-19) |
| Missing | 2 149 (23.3) | 703 (7.6) | 8 (8-9) |
| **Aspirin before breast cancer diagnosis** |  |  |  |
| No | 8 178 (88.6) | 2800 (30.3) | 12 (11-12) |
| Yes | 1 048 (11.4) | 347 (3.8) | 13 (11-14) |


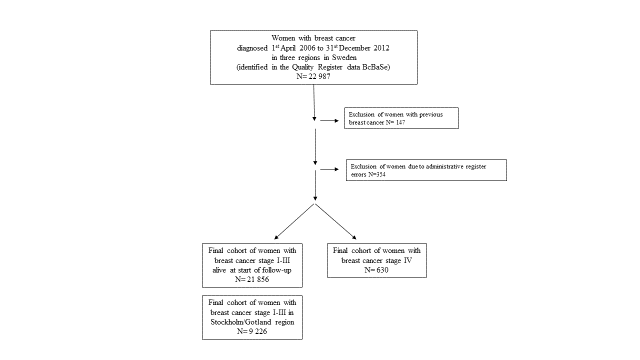
**Figure S1: Flow diagram of the included study participants**
